# Aerobic physical activity to improve memory and executive function in sedentary adults without cognitive impairment: A systematic review and meta-analysis

**DOI:** 10.1016/j.pmedr.2021.101496

**Published:** 2021-07-16

**Authors:** Coles M. Hoffmann, Megan E. Petrov, Rebecca E. Lee

**Affiliations:** a Arizona State University, Edson College of Nursing and Health Innovation, United States; b Barrow Neurological Institute, United States

**Keywords:** Physical activity, Aerobic, Cognitive function, Memory, Executive function, Adults

## Abstract

The worldwide population of adults ages 50 and older continues to increase and is projected to reach over 2.3 billion by 2030. Aging is the biggest risk factor for cognitive impairment and dementia. Aerobic physical activity may improve cognitive functioning, thus delaying aging-related cognitive decline.

The purpose of this review was to examine the effect of aerobic physical activity on memory and executive function in sedentary adults with no known cognitive impairment.

PubMed, CINAHL, Psycinfo, and Cochrane Library databases were systematically searched for peer-reviewed articles up to July 2019. Randomized controlled trials of sedentary adults, aged 50 and older, that compared an aerobic physical activity intervention to either no treatment or alternative active comparator and reported outcome measures of memory and/or executive function were included. A random effects meta-analysis was performed to examine the separate effect sizes for memory and executive function.

Nine studies met inclusion criteria and contributed either memory and/or executive function effect sizes (n = 547). Results from the random effects meta-analysis suggested, by post-intervention, a large effect size for the aerobic physical activity interventions on memory (g = 0.80, 95%CI: 0.14–1.47; n = 7; p = 0.02) and a small effect on executive function (g = 0.37, 95%CI: 0.04–0.69; n = 6; p = 0.03).

Aerobic physical activity may improve memory and executive function in sedentary adults without cognitive impairment. Policymakers and providers should promote aerobic physical activity in this population, and further research should investigate the most effective ways to promote aerobic physical activity in mid-life to older adults.

## Highlights

• Aerobic physical activity interventions had a large effect on memory.
• Aerobic physical activity interventions had a small effect on executive function.
• Meeting U.S. physical activity guidelines resulted in a large effect on memory.
• Studies with a no treatment control had a large effect on memory.

## 1 Introduction

In 2015, there were over 1.6 billion adults aged 50 and older, and that number is expected to increase to over 2.3 billion by 2030 (World Population Prospects, 2019). Cognitive decline is known to be associated with aging, especially from the age of 50 and above (Angevaren et al., 2008). As aging progresses, deterioration in a broad range of cognitive processes occurs, including decline in attention, processing speed, memory, and executive function (Hedden and Gabrieli, 2004, Royall et al., 2004, Yakhno et al., 2007).

Aerobic physical activity (PA), activity that leads to increased heart rate and more labored breathing (Piercy et al., 2018), may delay neurobiological and cognitive decline related to aging. Aerobic PA compared to other PA types may generate the largest improvements in memory and executive function (Colcombe et al., 2006, Erickson et al., 2011). However, the literature has reported mixed results. A meta-analysis of prospective cohort studies reported a significant relationship between PA, both low-to-moderate or high levels, and incident cognitive impairment in 33,816 “nondemented” adults (Sofi et al., 2011). Further, vigorous physical activity (e.g., aerobics, running, cycling) also was associated with prevention of dementia in later life. Similarly, in a meta-analysis of studies up to 2001, Colcombe and Kramer (2003) reported that aerobic PA interventions had robust effects on cognitive function, especially executive function in sedentary adults aged 55 and older (Colcombe and Kramer, 2003). In contrast, a systematic review and meta-analysis found aerobic PA compared to other active interventions (e.g., strength training, flexibility-enhancing) or to wait-list control groups had no effect on overall cognitive function (Young et al., 2015). This review noted that the internal validity and rigor (i.e., small sample sizes, important moderators were not analyzed) of the included studies was lacking. A limitation of many of the previous reviews examining aerobic PA and cognitive function is that many of the studies involve interventions that combine aerobic PA with other forms of PA (e.g. strength training), many included studies with interventions that did not meet US PA guidelines, and many did not look at specific domains of cognitive function.

The purpose of this systematic review and meta-analysis was to examine whether aerobic physical activity improves cognitive function, specifically memory and executive function, in sedentary adults (aged 50 + ) without cognitive impairment. It was hypothesized that aerobic physical activity interventions would result in a significant improvement in at least one measure of cognitive function (either memory, executive function, or both). The current review adds to the existing body of knowledge by including randomized controlled trials (RCTs) that were completed after the search limits of the Colcombe and Kramer (2003) review examining aerobic physical activity and cognition in “normal” sedentary adults. Prior studies, including Colcombe and Kramer (2003) have examined “aerobic PA,” but have included interventions that combined aerobic PA with other types of fitness training (e.g. strength training). The current review only includes studies with aerobic PA interventions that do not combine other types of PA, in order to isolate the true effect of aerobic PA from other types of PA. The current review consists of RCTs with active (e.g., stretching and strength training) comparators and no treatment control groups. The review focused solely on the domains of memory and executive function, as they have been identified as primary areas subject to decline as well as to improvement via physical activity (Colcombe et al., 2006, Erickson et al., 2011).

## 2 Methods

This systematic review was conducted and reported following the Cochrane Handbook for Systematic Reviews of Interventions (Higgins and Green, 2011).

### 2.1 Data sources and search strategy

PubMed, the Cumulative Index to Nursing and Allied Health Literature (CINAHL Plus), the Cochrane library, and PsycInfo were systematically searched for peer-reviewed articles published after the search limits of Colcombe and Kramer’s 2003 meta-analysis, from August 2001 to July 2019. A combination of MeSH and free text terms were used to find studies involving physical activity, and memory, and/or executive function in sedentary adults (50 + ). All possible search terms were entered into each search string, using the Boolean operators “AND” and “OR” to connect terms. The search string used to identify articles was exercise OR exercis* OR motor activit* OR physical activit* OR aerobic OR motor activit* AND cognit* OR memory OR memory episodic OR memory short-term OR memory long-term OR working memory OR mental process* OR executive function OR brain AND adult OR middle aged OR aged OR older OR old OR elderly OR geriatric AND sedentary OR underactive OR inactive. Titles, abstracts, and reference lists were screened to identify relevant articles and were examined in depth for inclusion and exclusion criteria.

### 2.2 Inclusion criteria and exclusion criteria

Studies were included if they met the following criteria: (1) Sedentary or inactive women or men aged 50 or older with no cognitive impairment. Therefore, studies with participants who were considered to have “mild cognitive impairment (MCI)” or dementia of any type were excluded from the review. (2) An aerobic PA program of any mode, duration, frequency, or intensity. If the aerobic PA intervention included other interventions (e.g. combined types of exercise training, combinations of aerobic exercise and mental training), then the study was excluded. (3) A control group that was either no treatment, or an alternative active treatment. (4) At least one outcome measure of memory or executive function measured at baseline and post-intervention, using a validated neuropsychological instrument. (5) The study design had to be a randomized controlled trial (RCT). (6) All peer-reviewed, published articles written in English.

### 2.3 Data extraction

The reviewer (CMH) screened the titles and abstracts of all of the studies that were identified by the search and eliminated duplicates and studies that unambiguously did not meet eligibility criteria. The remaining studies were examined in depth to extract eligibility criteria. Data on the main study characteristics including study population, study design, intervention, control, outcome measures, covariates, and main outcomes were recorded. Two independent reviewers (CMH and MEP) evaluated these study characteristics. The reviewers discussed any disagreements and a consensus was reached in all cases.

### 2.4 Risk of bias

The methodological quality of the included studies was assessed using the Cochrane Collaboration’s tool for assessing risk of bias (Higgins and Green, 2011). Areas examined for quality included seven methodological domains: random sequence generation, allocation concealment, blinding of participants and personnel, blinding of outcome assessment, incomplete outcome data, selective outcome reporting, and other sources of bias. Each domain included a series of yes or no questions to determine risk of bias. A judgement of “yes” indicates “low risk.” of bias. A judgement of “no” indicates “high risk,” or “unclear bias.”

### 2.5 Data synthesis

Articles were examined and grouped based on quality (risk of bias), study characteristics, and relationship between physical activity and cognitive function (memory, executive function, or both). The studies were also organized based on study location, number of participants, age of participants, and frequency (per week), duration (in minutes), and length (in weeks, months, or years) of the aerobic physical activity intervention. Last, the outcomes were grouped based on whether there was a significant outcome in memory and/or executive function and further organized based on whether the improvement occurred in memory, executive function, or both.

### 2.6 Meta-analysis

Pre and post intervention means and standard deviations were extracted from each study for either memory, executive function, or both, and transformed into mean differences and pooled standard deviations. Each article contributed either one or two effect sizes (memory, executive function, or both) to the meta-analysis. When multiple measures of memory and/or executive function were reported, the most common measure used across studies was selected (e.g. Logical Memory (delayed), Wisconsin Card Sorting Task (WCST)). Hedges *g*, a measure of effect size that corrects for the impact of small sample size and standard errors (Borenstein et al., 2009), was calculated for each sample. Meta-analyses were conducted using RevMan 5 (Manager, 2014), and a summary effect was provided based on a random effects meta-analysis for both memory and executive function. Heterogeneity was estimated using Cochran’s Q to determine if a random effects meta-analysis should be used to provide a better estimate among studies with high heterogeneity or low sample sizes. Forest plots were created using adjusted effect sizes and their 95% confidence intervals. To examine potential explanations for heterogeneity, sub-analyses were conducted to compare studies across the following characteristics: aerobic physical activity interventions that met U.S. physical activity guidelines (≥150 min per week) vs. those that did; no treatment control vs. active control; short or long in duration (≤6 months vs ≥ 6 months); and country of origin (in the U.S. vs outside of the U.S.). A p-value of < 0.05 was considered significant for the primary meta-analysis and all sub-analyses.

## 3 Results

**Fig. 1:**
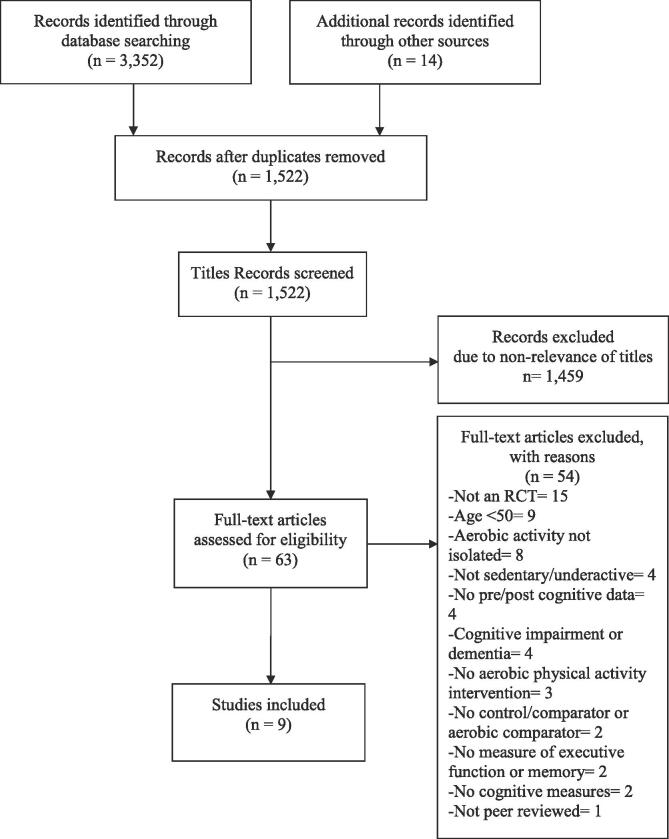
PRISMA (Preferred Reporting Items for Systematic Reviews and Meta-Analysis) flow diagram of study selection process (Moher et al., 2009).

Overall, 3,352 articles were identified through database searches and 14 additional studies were added after reviewing references from the relevant literature (see Fig. 1). After individual titles were reviewed and duplicates were removed, 63 studies remained. After abstracts, titles, and full texts were examined, nine articles were included in this review (see Fig. 1).

### 3.1 Risk of bias

The risk of bias for each domain in each study (low risk, unclear risk, or high risk) was assessed and is presented in Table 1. All nine included studies were judged as low risk in the domains of random sequence generation and other sources of bias. A majority of the studies were low risk for selective outcome reporting (8 studies) (Erickson et al., 2011, Albinet et al., 2010, Ruscheweyh et al., 2011, Voss et al., 2013, Chapman et al., 2013, Antunes et al., 2015, Antunes et al., 2015, Albinet et al., 2016) and incomplete outcome data (6 studies) (Erickson et al., 2011, Albinet et al., 2010, Ruscheweyh et al., 2011, Chapman et al., 2013, Antunes et al., 2015, Antunes et al., 2015, Vidoni et al., 2015). A majority of the studies were judged to have unclear risk of allocation concealment (7 studies) (Erickson et al., 2011, Albinet et al., 2010, Ruscheweyh et al., 2011, Voss et al., 2013, Chapman et al., 2013, Antunes et al., 2015, Albinet et al., 2016) and blinding of outcome assessors (6 studies) (Erickson et al., 2011, Albinet et al., 2010, Voss et al., 2013, Chapman et al., 2013, Antunes et al., 2015, Albinet et al., 2016). The remaining studies were judged as low risk in these domains. All of the studies were deemed high risk for blinding of participants and trainers due to the nature of the intervention.

**Table 1:** Risk of bias in included studies in accordance with Cochrane Collaboration Guidelines.

Study	Random sequence generation	Allocation concealment	Blinding (participants and trainers)	Blinding (outcome assessors)	Incomplete outcome data	Selective outcome reporting	Other sources of bias
Albinet et al., 2010	+	?	–	?	+	+	+
Erickson et al., 2011	+	?	–	?	+	+	+
Ruscheweyh et al., 2011	+	?	–	+	+	+	+
Voss et al., 2013	+	?	–	?	–	+	+
Chapman et al., 2013	+	?	–	?	+	+	+
Vidoni et al., 2015	+	+	–	+	+	_	+
Antunes et al., 2015a	+	+	–	+	+	+	+
Antunes et al., 2015b	+	?	–	?	?	+	+
Albinet et al., 2016	+	?	–	?	–	+	+

Footnote: +=low risk; -=high risk; ?=unclear.

### 3.2 Study characteristics

All nine studies contributed effect size data for either memory (Erickson et al., 2011, Ruscheweyh et al., 2011, Voss et al., 2013, Chapman et al., 2013, Antunes et al., 2015, Antunes et al., 2015, Vidoni et al., 2015) and/or executive function (Albinet et al., 2010, Voss et al., 2013, Chapman et al., 2013, Antunes et al., 2015, Albinet et al., 2016, Vidoni et al., 2015). Four out of nine of the studies were conducted in the United States [7, 15, 16, 20], one in Germany (Ruscheweyh et al., 2011), two in France (Albinet et al., 2010, Albinet et al., 2016), and two in Brazil (Antunes et al., 2015, Antunes et al., 2015) (Table 2). Seven of the nine studies had<75 participants (Albinet et al., 2010, Ruscheweyh et al., 2011, Voss et al., 2013, Chapman et al., 2013, Antunes et al., 2015, Antunes et al., 2015, Albinet et al., 2016), and the remaining two studies had between 100 and 120 participants (Erickson et al., 2011, Vidoni et al., 2015). Sedentary behavior was defined differently across studies, including being physically active for no more than 30 min total in the past 3–6 months (Erickson et al., 2011, Albinet et al., 2010, Voss et al., 2013, Chapman et al., 2013, Antunes et al., 2015, Albinet et al., 2016, Vidoni et al., 2015), not participating in physical activity for 20–30 min more than two times per week (Ruscheweyh et al., 2011, Chapman et al., 2013), and based on the evaluation of aerobic capacity (Antunes et al., 2015).

**Table 2:** Summary of study characteristics of included studies.

Author	Country	Subjectsn	Gender, M/F	Age, range	Intervention(s), control/comparator	Duration/Frequency/Length	Executive Function and/or Memory Assessment^a^
Albinet et al., 2010	France	24	11/13	65–78	1. Aerobic exercise (walking, running) 2. Stretching control	3xwk/60 min/3 months	-Wisconsin Card Sorting Task (WCST)
Erickson et al., 2011	USA	120	40/80	55–80	1. Aerobic exercise (walking) 2. Stretching control	1xwk/40 min/1 year	-Spatial Memory Paradigm Task
Ruscheweyh et al., 2011	Germany	62	22/40	50–72	1. Nordic Walking 2. Gymnastics 3. No treatment control	3xwk/50 min/6 months	-Auditory Verbal Learning Test (AVLT)
Voss et al., 2013	USA	70	25/45	55–80	1. Aerobic walking 2. Flexibility, toning, and balance control	3xwk/40 min/1 year	-Wisconsin Card Sorting Task (WCST)-Digit Span forward
Chapman et al., 2013	USA	37	10/27	57–75	1. Aerobic exercise (bike and treadmill) 2. Wait-list control	3xwk/60 min/3 months	-The Stroop Task-Logical Memory (LM) delayed
Vidoni et al., 2015	USA	101	36/65	66–78	1. 75 min aerobic exercise 2. 150 min aerobic exercise 3. 225 min aerobic exercise 4. No treatment control	75 min-225 min per wk/26 wks	-The Stroop task-Logical Memory (LM) delayed
Antunes et al., 2015a	Brazil	51	0/51	60–70	1. Aerobic exercise 2. Leisure 3. No treatment control	3xwk/60 min/6 months	-Wisconsin Card Sorting Task (WCST)-Logical Memory (LM) delayed
Antunes et al., 2015b	Brazil	46	46/0	60–75	1. Aerobic exercise (cycle ergometer) 2. No treatment control	3xwk/60 min/6 months	-Free Word Recall Test
Albinet et al., 2016	France	36	10/26	60–75	1. Aquaerobics and swimming 2. Stretching control	2xwk/60 min/21 weeks	-The Stroop Task

a Assesments used in the *meta*-analysis.

Three of the studies examined the association between aerobic physical activity and memory only (Erickson et al., 2011, Ruscheweyh et al., 2011, Antunes et al., 2015), two examined the association between aerobic physical activity and executive function only (Albinet et al., 2010, Albinet et al., 2016) and the remaining four examined the association between aerobic physical activity and both memory and executive function (Voss et al., 2013, Chapman et al., 2013, Antunes et al., 2015, Vidoni et al., 2015). Intervention length in these trials ranged from three months to one year. Intervention duration ranged from 40 to 60 min per session and intervention frequency ranged from one to three times per week. Two out of nine studies employed a short-term intervention (three months) (Albinet et al., 2010, Chapman et al., 2013), five studies employed a five to seven-month intervention (Ruscheweyh et al., 2011, Antunes et al., 2015, Antunes et al., 2015, Albinet et al., 2016, Vidoni et al., 2015), and the remaining two studies employed a long term intervention (one year) (Erickson et al., 2011, Voss et al., 2013). Five of the studies included a no treatment control group (Ruscheweyh et al., 2011, Chapman et al., 2013, Antunes et al., 2015, Antunes et al., 2015, Vidoni et al., 2015), and the remaining four used another type of exercise training for the control group (e.g. stretch, balance) (Erickson et al., 2011, Albinet et al., 2010, Voss et al., 2013, Albinet et al., 2016).

Aerobic physical activity types varied (e.g., brisk walking, jogging, cycling, or swimming) and were either directed by a trained exercise instructor or independently-led activities. Equipment used for the aerobic physical activity interventions included treadmills and bicycle ergometers. All active control groups were led by an exercise instructor.

All studies assessed cognitive function with objective measures using validated neuropsychological assessments. Several of the studies utilized immediate and delayed Logical Memory (LM) and immediate and delayed word recall. Other measures of memory utilized include digit span forward and a computerized spatial memory task. All of the studies that measured executive function utilized the Stroop Task, and/or the Wisconsin Card Sorting Test (WCST).

### 3.3 Random effects meta-analysis

**Fig. 2:**
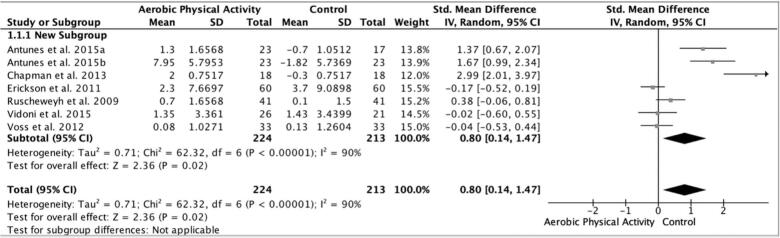
Forest plot of the effect sizes for aerobic physical activity interventions on memory domain (random effects meta-analysis). Review conducted in August 2019.

3.3.1 Memory. Results from the random effects meta-analysis suggested a large effect size for the aerobic physical activity interventions on memory at post-intervention (g = 0.80, 95%CI: 0.14–1.47; n = 7; p = 0.02) (see Fig. 2). Due to high heterogeneity between the study effects (Cochran’s Q = 62.32, p < 0.00001), subgroup analyses were conducted to explore differences (See Table 3). There were several significant effect sizes among the sub-group analyses, including a significant and larger effect size for studies that met U.S. physical activity guidelines (g = 1.21, 95%CI: 0.32–2.11; n = 5; p = 0.008) (Ruscheweyh et al., 2011, Chapman et al., 2013, Antunes et al., 2015, Antunes et al., 2015, Vidoni et al., 2015), the study that was<6 months in length (g = 2.99, 95%CI: 2.01–3.97; p=<0.00001) (Chapman et al., 2013), studies that had a no treatment control (g = 1.21, 95%CI: 0.32–2.11; n = 5; p = 0.008) (Ruscheweyh et al., 2011, Chapman et al., 2013, Antunes et al., 2015, Antunes et al., 2015, Vidoni et al., 2015) and studies that took place outside of the U.S. (g = 1.10, 95%CI: 0.25–1.95; n = 3; p = 0.01) (Ruscheweyh et al., 2011, Antunes et al., 2015, Antunes et al., 2015). None of the sub-analyses completely explained the heterogeneity of the results for memory.

**Table 3:** Sub-Analyses to Explore Differences in Memory Domain (n = 7).

	n	Hedges *g* (95% C.I.)	p
**U.S. Physical Activity Guidelines**			
Meeting	5	**1.21 (0.32–2.11)**	**0.008**
Not meeting	2	−0.12 (−0.41–0.17)	0.41
**Study Length**			
<6 months	1	**2.99 (2.01–3.97)**	**<0.00001**
≥6 months	6	0.48 (−0.06–1.03)	0.08
**Control group**			
No treatment	5	1.21 (0.32–2.11)	0.008
Active control	2	−0.12 (−0.41–0.17)	0.41
**Country**			
Outside of the U.S.	3	**1.10 (0.25–1.95)**	**0.01**
U.S.	4	0.58 (−0.35–1.51)	0.22
**Memory Assessment**			
Logical Memory (LM)	3	1.40 (−0.21–3.02)	0.09
Word Recall	2	0.99 (−0.27–2.26)	0.12
Digit Span forward	1	−0.04 (−0.53–0.44)	0.86
Spatial Memory Task	1	−0.17 (−0.52–0.19)	0.37

**Fig. 3:**
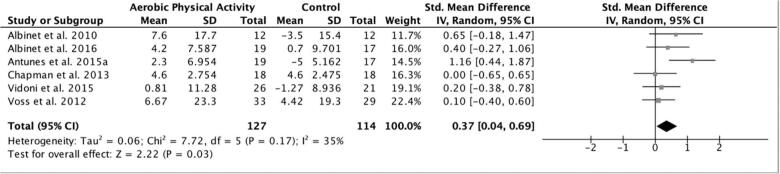
Forest plot of the effect sizes for aerobic physical activity interventions on executive function domain (random effects meta-analysis). Review conducted in August 2019.

3.3.2 Executive function. Results from the random effects meta-analysis suggested a small effect size for the aerobic physical activity interventions on executive function at post-intervention (g = 0.37, 95%CI: 0.04–0.69; n = 6; p = 0.03) (see Fig. 3). Heterogeneity was not significant (Cochran’s Q = 7.72, p < 0.17), therefore subgroup analyses are not reported here, but can be found in Table 4. Sub-group analyses that involved only one study as a comparator were examined for consistency and are indicated in Table 4.

**Table 4:** Sub-Analyses to Explore Differences in Executive Function Domain (n = 6).

	n	Hedges *g* (95% C.I.)	p
**U.S. Physical Activity Guidelines**			
Meeting	5	**0.45 (0.06–0.83)**	**0.02**
Not meeting	1	0.10 (−0.40–0.60)	0.69
**Study Length**			
<6 months	3	0.44 (−0.15–1.04)	0.15
≥6 months	3	0.30 (−0.10–0.71)	0.14
**Control group**			
No treatment	3	0.43 (−0.23–1.09)	0.20
Active control	3	0.29 (−0.07–0.65)	0.11
**Country**			
Outside of the U.S.	3	**0.72 (0.27–1.18)**	**0.002**
U.S.	3	0.11 (−0.22–0.43)	0.52
**Executive Function Assessment**			
Wisconsin Card Sorting Test (WCST)	3	0.59 (−0.06–1.25)	0.08
Stroop Task	3	0.20 (−0.17–0.56)	0.29

## 4 Discussion

Overall, this systematic review and meta-analysis found that aerobic physical activity interventions result in a significant improvement in at least one measure of cognitive function (either memory, executive function, or both) in adults aged 50 and up, supporting our hypothesis. These echo the results from a similar meta-analysis performed by Colcombe and Kramer (2003) that reported aerobic PA interventions had a significant effect on cognitive function, especially executive function, in adults aged 55 and up. While Colcombe and Kramer (2003) found a medium effect size for executive function, this review yielded a small, but significant effect on executive function. In addition to the previous meta-analysis, this review found that aerobic PA had a large effect on memory. This meta-analysis expanded on Colcombe and Kramer’s (2003) original meta-analysis by including a larger age range, examining memory in addition to executive function, and by including interventions that only had aerobic PA interventions (as opposed to mixed interventions).

Several meta-analytic studies and systematic reviews examining similar hypotheses have been published over the past 20 years. There are mixed findings among many of the existing reviews examining improvement in cognitive function in “normal” adults due to physical activity. Two previous meta-analyses found that across a variety of study designs (e.g. RCT, cross-sectional), age ranges, and patient populations there was a small effect of PA interventions on cognitive function, including memory and executive function (Etnier et al., 1997, Van Uffelen et al., 2008). In a meta-analysis that included young adults and adults with mild cognitive impairment, researchers found an overall small, but significant effect of PA on cognition (Etnier et al., 1997). In contrast, a meta-analysis that included 12 RCTs that examined the effect of aerobic PA on cognitive function in cognitively normal adults aged 55 and up, reported no cognitive benefit from aerobic PA (Young et al., 2015). One limitation of the last review was that it only included studies that had a measure of cardiorespiratory fitness and many of the interventions included more than just aerobic exercise.

Sub-analyses that were performed to understand the high heterogeneity in the effect size for the memory domain illuminated some important results. First, sub-analyses showed that studies that met US PA guidelines had a significant and large effect compared to studies that did not meet guidelines. Previous research has examined effect by program duration (1–6 + months), session duration (15–60 min), and have found that moderate and long session duration yield medium and low effect sizes, respectively (Colcombe and Kramer, 2003), but none have examined the effect of meeting U.S. PA guidelines versus not meeting guidelines. Second, sub-analyses revealed that the study that was<6 months in length had a significant and large effect size, compared to studies that were 6 months or more. This result was in contrast to Colcombe and Kramer’s (2003) review, which found that studies with interventions lasting more than 6 months had a medium effect size that was larger than both the short (1–3 months) and medium (4–6 months) program duration. This result was based on one study that was<6 months and should therefore be interpreted with caution. Third, studies that had no treatment control yielded a significant and large effect compared to studies with an active control. This finding appears intuitive, as previous research has shown that other types of PA (e.g. strength training), may improve cognitive function (Van Uffelen et al., 2008); therefore, no treatment controls may be able to better detect effects of aerobic PA interventions. Last, sub-analyses revealed that studies that took place outside of the U.S. had a significant and large effect, compared to studies in the U.S. Interestingly, this result may be explained by the fact that all of the studies outside of the U.S. that contributed an effect size for memory implemented interventions that met the U.S. PA guidelines.

Overall, sub-analyses from this study should be viewed as exploratory and interpreted with caution, as they were developed in attempt to explain heterogeneity and were not created a priori. Due to the small number of studies, some of the sub-analyses included only one study in the subgroup (e.g. < 6 months program duration), therefore the study represents an effect from that single study rather than an overall effect.

There are a number of major mechanisms by which aerobic PA is thought to improve memory and executive function. First, aerobic PA has been linked with gray and white matter volume increases in the temporal and prefrontal cortices, as well as hippocampal volume, which are specifically associated with long-term memory and executive function, respectively, as well as dementia and AD collectively (Colcombe et al., 2006). In further support, another RCT found that aerobic PA increased hippocampal volume, and also found improvement in memory function (Erickson et al., 2011). According to Erickson et al. (2011), aerobic PA may improve areas that tend to show the greatest decline in aging adults (e.g. prefrontal cortex and hippocampus). Both memory and executive function tend to decline the most with normal aging, MCI, and AD (Kirova et al., 2015). A second mechanistic pathway is that aerobic PA may improve cognitive function through increases in brain-derived neurotrophic factor (BDNF), insulin-like growth factor 1 (IGF 1) and increased cerebral blood volume (CBV) (Vaynman et al., 2004, Carro et al., 2001, Pereira et al., 2007). Previous research has asserted that the relationship between aerobic PA and cognitive function may be mediated by improved cardiovascular (aerobic) fitness, and several studies included in this review reported improved cardiovascular fitness (Young et al., 2015, Chapman et al., 2013, Antunes et al., 2015, Antunes et al., 2015). What is unknown is whether aerobic PA, through these proposed mechanisms, has a true differential effect on different cognitive domains like that which was demonstrated in the present meta-analysis (i.e., a large effect for memory and a small effect for executive function). Future research should examine both the relative impact of aerobic PA on multiple cognitive domains and on these potential neurological underpinnings. Future research should also examine the relationship between cardiovascular fitness and cognitive function.

A strength of this meta-analysis was that it included only RCTs which are considered the highest quality studies. A second strength is that it examined studies that included aerobic PA interventions that were not mixed with other types of PA. By selecting only studies with strictly aerobic PA interventions, the review can specifically analyze the effect of aerobic PA on memory and executive function. A final strength of both this study, and of the conclusions that can be drawn from it, is that more than half of the studies included in this review had an overall low risk of bias.

Nevertheless, the studies examined in this review had several limitations impacting the quality of the evidence, and the conclusions that may be drawn from this review. First, the sample size for many of the studies was low, with seven out of nine of the studies having<75 participants (Albinet et al., 2010, Ruscheweyh et al., 2011, Voss et al., 2013, Chapman et al., 2013, Antunes et al., 2015, Antunes et al., 2015, Albinet et al., 2016). Several of the assessments included in the studies (e.g. Logical Memory delayed (Chapman et al., 2013, Antunes et al., 2015, Vidoni et al., 2015), Wisconsin Card Sorting Test (Albinet et al., 2010, Voss et al., 2013, Antunes et al., 2015) are subject to practice effects, or familiarization of previous cognitive assessments, which can lead to better post-test scores, and inflated effect sizes. None of the studies utilized a set-shifting task as a measure of executive function, which may be more sensitive to change in PA interventions. The use of these assessments and possible practice effects may account for the large effect size for memory and small effect size for executive function observed in this meta-analysis relative to Colcombe and Kramer’s (2003) reported large effect for executive function. The current and prior meta-analyses remain plagued by a lack of comprehensive neuropsychological batteries, which may explain the differences in effect sizes between studies. Another limitation of the existing research was that three of the studies did not meet the U.S. Department of Health and Human Services recommended guidelines for aerobic PA for adults (at least 150 min of moderate PA per week or 75 min of vigorous PA per week) (Piercy et al., 2018). All of the studies lacked long term follow-up in order to examine whether significant changes in cognitive function were maintained. Several of the studies also lacked representativeness due to exclusion criteria which excluded many adults on the basis of certain diseases or disorders, such as cancers, heart diseases, diabetes, and depression symptomatology. Several of the studies also lacked equal sex distribution, with women making up over 50% of the sample in eight out of nine studies. Colcombe and Kramer (2003) found that studies with more women yielded greater effect sizes for improvement in cognitive function as a result of PA interventions, therefore future studies should examine the potential moderating effect of sex. A final limitation is that many of the studies used different criteria or failed to clearly define how they measured “sedentary” behavior likely due to the lack of clear clinical cut-offs for “sedentary” behavior.

This review indicates aerobic PA alone can improve sedentary adults’ cognitive abilities, in executive function and memory. However, the sustainability of this effect is unknown. Future RCTs will need to assess cognitive functioning over long-term follow-up. Future research also should focus on assessing the dose–response relationship of this effect, testing interventions that meet recommended PA guidelines, and examining sociobehavioral and physiological mechanisms of the relationship between aerobic PA and improved cognitive function (e.g. socialization, aerobic fitness capacity). Larger trials employing more consistent and sensitive batteries of neuropsychological assessments are needed for increased rigor and ability to compare results across studies. Most prior studies did not include representative or diverse samples or did not report these characteristics making generalizability of the results difficult to assess. Future studies would benefit from inclusion of a higher proportion of men and diversity in race/ethnicity and included comorbidities.

The implications of the present review’s results are profound considering that aerobic PA is a modifiable lifestyle factor with high potential for delaying or preventing the onset of cognitive impairment and dementia. Additionally, the review reinforces the importance of meeting the minimum US PA guidelines, with evidence from several RCTs. The prevention of cognitive impairment of adults has important individual, healthcare, and economic implications. Of particular importance is the inclusion of implementation strategies and behavior change techniques (e.g. self-reward, graded tasks) to PA intervention, both of which may increase or sustain PA in adults, to prevent devastating and costly conditions later in life (Howlett et al., 2018).

## Funding Sources

None

Human Rights: All procedures performed in studies involving human participants were in accordance with the ethical standards of the institutional and/or national research committee and with the 1964 Helsinki declaration and its later amendments or comparable ethical standards.

Informed Consent: Informed consent was obtained from all individual participants included in the study.

## Declaration of Competing Interest

The authors declare that they have no known competing financial interests or personal relationships that could have appeared to influence the work reported in this paper.
